# Carbon dioxide stunning of pigs induces the expression of fear-associated genes in the amygdala

**DOI:** 10.1038/s41598-026-51710-9

**Published:** 2026-05-06

**Authors:** Julia Gelhausen, Nora-F. Paul, Jonas Knöll, Inga Wilk, Daniel Mörlein, Jens Tetens, Clemens Falker-Gieske

**Affiliations:** 1 Department of Animal Sciences, Georg-August-University, Burckhardtweg 2, 37077 Göttingen, Germany; 2 Institute of Animal Welfare and Animal Husbandry, Friedrich-Loeffler-Institute, Dörnbergstr. 25/27, 29223 Celle, Germany; 3 Center for Integrated Breeding Research, Georg-August-University, Albrecht-Thaer-Weg 3, 37075 Göttingen, Germany

**Keywords:** CO_2_, Amygdala, Nitrogen, Argon, Pig, Animal welfare, Stunning, Amygdala, Gene expression, Animal behaviour

## Abstract

Stunning pigs with inert gases induces less aversive behavior before loss of consciousness than stunning with high-concentration carbon dioxide (CO_2_). As the amygdala is the center of emotional processing, we hypothesized to find differences in the amygdala transcriptome depending on the gas used for stunning. Therefore, 27 amygdala samples were collected from pigs that were stunned with either argon (Ar), a mixture of nitrogen (N_2_) and Ar, or CO_2_. From each sample, total RNA was isolated and sequenced. Compared with inert gases, CO_2_ stunning induced an amygdala transcriptional profile consistent with increased fear-related processing. For example, gene set enrichment analysis identified *G protein‑coupled serotonin receptor activity* as significantly enriched only in comparisons between CO_2_ stunning and inert‑gas stunning. In this context, *HTR1A* and *HTR2A*, which encode serotonin receptors, were downregulated in the animals stunned with CO_2_. Both receptors have already been described to be involved in psychological disorders and fear, suggesting that CO_2_ stunning induces more fear-like emotions. Together, these transcriptomic differences support previous reports of more aversive responses during CO_2_ stunning. This study provides the first whole‑transcriptome analysis of porcine amygdalae following stunning with different gas mixtures and highlights candidate transcripts for benchmarking welfare outcomes of stunning gases.

**Supplementary Information:**

The online version contains supplementary material available at 10.1038/s41598-026-51710-9.

## Introduction

The formation of fear, anxiety, and aversion is based on a complex network of innate and learned reactions. Researchers have used animal models to understand these mechanisms by observation and interpretation of innate or conditioned behavior, such as the innate avoidance of bright and open areas of mice to protect themselves from predators. In combination with a targeted knockout of the brain areas of interest, predictions of function regarding the specific regions can be made^1^. In case of pig husbandry, behavior has been the subject of several studies to investigate animal welfare during several production stages from weaning to fattening^2^, including the analysis of gene expression in different brain regions of socially stressed pigs^3^. As slaughter remains to be the last step in the life of a fattening pig, animal welfare during this process has to be on the highest possible level, which includes the time of transport as well as stunning and killing. According to the Council Regulation (EC) No 1099/2009^4^, animals must be stunned in such a way that the loss of consciousness and insensibility is maintained until death by exsanguination. For pigs, two methods are permitted under commercial conditions: electrical and gas stunning. Electrical stunning induces unconsciousness by affecting the electrical activity of the neurons via an external current source, as reviewed in Terlouw, Bourguet & Deiss^5^. While the onset of unconsciousness is quick in comparison to gas stunning systems, the correct placement of the electrodes with the correct current is crucial for successful stunning and animal welfare^6^. Unlike electrical stunning, high-concentration CO_2_ stunning works by lowering gondolas with a group of pigs into a pit with a CO_2_ atmosphere of at least 80%^4,5^. High concentrations of CO_2_ in the breathing air interfere with the exchange of CO_2_ within the lungs. As a consequence, partial pressure of CO_2_ (pCO_2_) increases, combined with a pH drop in the blood^7^. As CO_2_ passes the blood–brain barrier, the pH of the cerebrospinal fluid is also decreased^5^ which results in a dysfunction of the brain cells, as the innervation of neurons is pH dependent^8^. This induces a deep unconsciousness, which can persist long enough to keep the animals unconscious until death. Even though CO_2_ stunning is very efficient, the initial exposure to the CO_2_ atmosphere poses a critical problem, as pigs show aversive behavior like gasping, hyperventilation or flight attempts^9–11^. Hyperventilation occurs due to the detection of the reduced pH by chemoreceptors in the medulla^12,13^, leading to an increase of ventilation to maintain homeostasis^14^. Additionally, the formation of carbonic acid in the nasal mucosa induces pain^15^. It is therefore essential to find alternatives that induce less aversive behavior while retaining the advantage of group stunning. In this context, the inert gases Argon (Ar) and Nitrogen (N_2_) have been the subject of previous studies^9,10^. Inert gas stunning acts via the induction of hypoxia. As the brain is dependent on oxygen (O_2_), a lack of O_2_ causes neurons to switch to anaerobic metabolism, resulting in an acidification of the extra- and intracellular fluid of the neurons^16^. Although, hypoxia can be life-threatening, especially as the brain is most dependent on oxygen supply, pigs that are exposed to inert gases show less aversive behavior than those that are stunned with high concentrations of CO_2_^9,10^. This difference in behavior is attributed to the fact that an increase in pCO_2_, rather than the absence of O_2_, is the primary physiological trigger of respiration^13^. As the amygdala is known to be the center of fear processing, it is likely that this brain area is involved in the different reactions to hypoxia and hypercapnia. A direct connection between acid-sensing ion-channel-1a (ASIC1a) in the amygdala and fear behavior in mice was shown by Ziemann et al*.*^17^. However, up to now, little is known about the mechanisms affected in the pig amygdala throughout stunning. Therefore, this study aimed to characterize the amygdala transcriptome and associated molecular biological pathways during inert gas and high concentration CO_2_ stunning. The gene expression profiles are biomarkers that may assist in the selection of the stunning gas composition that provides the highest level of animal welfare.

## Results

To potentially improve animal welfare, we sought to identify transcriptomic signatures in the amygdala pointing to the stunning gas composition, which is the least stressful for pigs prior to slaughter. For that purpose, the amygdalae of 27 pigs were isolated after stunning with three different gas mixtures, followed by slaughtering, and subjected to RNA sequencing (RNAseq). By comparing gene expression in the amygdalae between pigs, which were stunned with Ar, CO_2_, or N_2_, we discovered different numbers of differentially expressed genes (DEGs) (Table 1, Supplementary Information S1).

**Table 1:** Summary of differential gene expression results in the amygdalae from pigs stunned with Ar, CO_2_, or N_2_ prior to slaughtering (abs. Log_2_ FC > 1).

	Ar vs CO_2_	Ar vs N_2_	CO_2_ vs N_2_
P < 0.01	95	30	172
P < 0.05	265	111	317

**Fig. 1:**
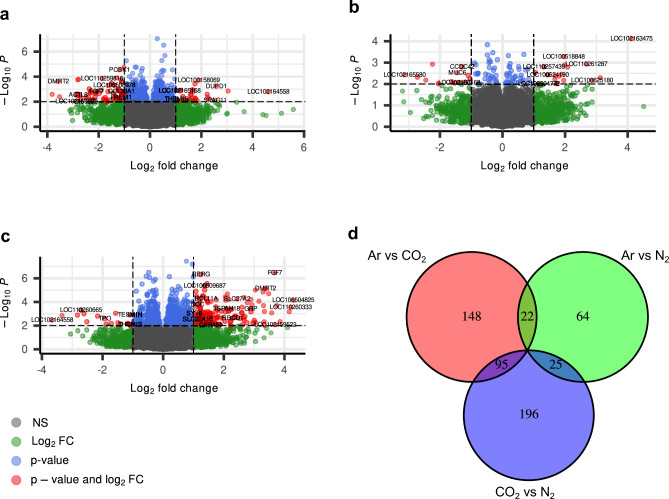
Volcano Plots of differential gene expression in the amygdalae of pigs stunned with different gas mixtures. (**a**) Ar vs CO_2_, (**b**) Ar vs N_2_, (**c**) CO_2_ vs N_2_ (significance thresholds: abs. Log_2_ FC > 1, p < 0.01). Gray datapoints indicated no statistical significance, green datapoints indicate significant Log_2_ FC, blue datapoints indicate significant p-value, and red datapoints indicate significant Log_2_ FC and p-value, respectively. (**d**) Venn Diagram of concordant and discordant differentially expressed genes between the group comparisons (significance thresholds: abs. Log_2_ FC > 1, p < 0.05).

**Fig. 2:**
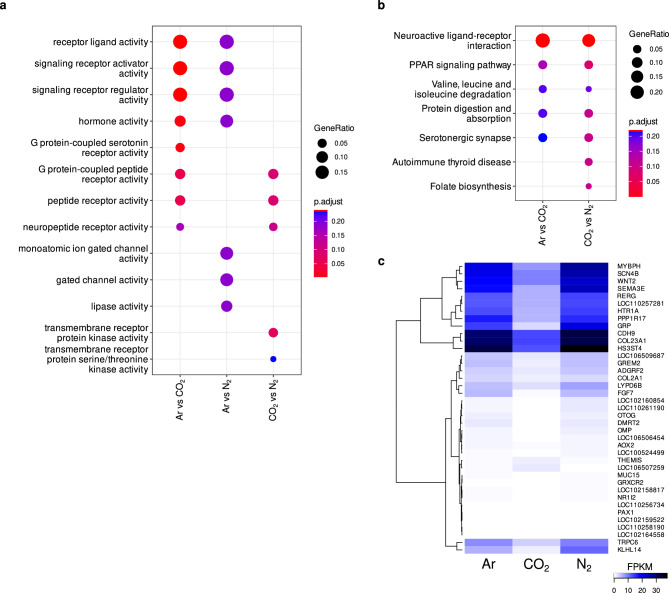
Gene set enrichment analysis results computed with clusterProfiler for (**a**) gene ontology molecular functions and (**b**) KEGG pathways (q-value > 0.25). (**c**) Heatmap of average FPKM values of differentially expressed genes (significance thresholds: abs. Log_2_ FC > 1, p < 0.01), which were common in the group comparisons Ar vs CO_2_ and CO_2_ vs N_2_, to show the effects that exposure to CO_2_ had on gene expression.

Stunning with CO_2_ had the strongest effect on gene expression in the amygdala (Fig. 1a and c) showing little overlap with the differential gene expression analysis of Ar vs N_2_ treatment (Fig. 1b and d). The largest overlap between DEGs was detected between the two comparisons in which Ar and N_2_ were each compared to CO_2_ stunning (Fig. 1d, Supplementary Information S2). Among those 95 overlapping genes were the anxiety-associated genes *GAL*^18^, *HTR1A* and *HTR2A*^19^. The latter two are serotonin receptors, which is a major neurotransmitter in anxiety^20^. In our gene set enrichment analysis (GSEA) serotonin related pathways were only discovered in the group comparisons involving CO_2_ (Fig. 2a and b, Supplementary Information S3) with the associated genes being *HTR2A*, *HTR1F*, and *HTR1A3* for the GO molecular function *G protein-coupled serotonin receptor activity* and *TPH2*, *HTR2A*, *CYP2C49*, *SLC18A2*, *HTR1A*, *LOC102163598*, and *HTR1F* for the KEGG pathway *Serotonergic synapse*. Several solute carrier (SLC) proteins have been linked to anxiety disorders, in particular the serotonin transporter gene *SLC6A4*^21^. In the comparison of CO_2_ vs N_2_, eleven SLC genes were upregulated in the CO_2_ group (Table 2).

**Table 2:** Solute carrier (SLC) proteins, which were significantly upregulated in the amygdalae of stunned pigs in the group comparison CO_2_ vs N_2_.

Gene	Substrate	Log_2_ FC
*SLC13A2*	sodium-dicarboxylate^51^	1.74
*SLC17A8*	glutamate^52^	1.23
*SLC18A2*	monoamine^53^	1.47
*SLC22A1*	organic cation^54^	2.17
*SLC22A16*	organic cation / carnitine^55^	1.14
*SLC27A2*	fatty acid^56^	2.44
*SLC34A2*	sodium phosphate^57^	1.50
*SLC36A3*	amino acids^58^	1.08
*SLC5A7*	choline^59^	2.06
*SLC6A3*	dopamine^60^	1.60
*SLC9A4*	potassium / sodium^61^	2.11

To visualize the most pronounced effects that exposure to CO_2_ had on gene expression, we constructed a heatmap of common highly significant DEGs (p < 0.01) of the two CO_2_ comparisons and plotted the average fragments per kilobase of transcript per million fragments mapped (FPKM) values of each experimental group (Fig. 2c, Supplementary Information S4). This revealed mostly uniform expression patterns between Ar and N_2_ treatment, which deviated strongly from CO_2_ treatment, where expression for all the visualized genes was lower. The most striking differences were observed between the twelve upper and the two lower genes in the heatmap. Protein–protein interaction (PPI) analyses with highly significant DEGs (p < 0.01) revealed more interactions as expected (p = 1.89 × 10^–6^) between the DEGs derived from the CO_2_ vs N_2_ comparison (Supplementary Information S5) with the largest cluster consisting of multiple SLC proteins as well as *HTR1A* and *HTR2A*, confirming the relevance of these genes in the neurophysiological response to CO_2_ stunning. To have a reference dataset for fear-related gene expression, we remapped RNAseq data that was derived from the amygdalae of fear-conditioned mice^22^ to the most recent *Mus musculus* reference genome (Supplementary Information S6). We discovered that the genes *DRD5*, *KRT27*, *SATB2*, *TH*, and *ZAR1* were commonly differentially expressed with the DEGs between CO_2_ vs N_2_ treatment.

## Discussion

The increase of CO_2_ in the breathing air has been coupled with feelings of fear and panic in humans^23^. Because increasing pCO_2_ and a decreasing pH in the blood and cerebrospinal fluid can be life-threatening, fear behavior is linked to these events. As the amygdala is responsible for emotional responses, we assumed to find differences in the amygdala transcriptome^24^. As CO_2_ stunning has been criticized to induce aversive behavior in pigs^10^, we hypothesized to find stress, fear or anxiety-related genes to be affected by the use of CO_2_ for stunning prior to slaughter. The study presented here is the first to compare the amygdala transcriptomes of pigs exposed to different stunning gases.

Comparison of the amygdala transcriptomes showed the highest count of significant DEGs for the comparisons of CO_2_ with the inert gases (Table 1), indicating that the stunning by hypercapnia induces different transcription profiles in this brain area. Our analysis showed that several genes that are related to processing and response to fear and anxiety were differentially expressed by the different gas mixtures used for stunning. Especially genes that are related to serotonin pathways (*G protein-coupled serotonin receptor activity & Serotonergic synapse,* Fig. 2a and b, Supplementary Information S3) were altered in the CO_2_ group. It is known that serotonergic neurons are involved in sensing changes in pH and carbon dioxide^25^. In addition, the serotonin system is associated with several psychological disorders in humans, such as panic disorder^26^ and anxiety^24,27^. In our data, the gene expression (*HTR1A* & *HTR2A*) of two serotonin receptors (5-HT_1A_ and 5-HT_2A_) was downregulated in the amygdalae of the pigs, which were stunned with CO_2_. Both receptors have been intensively investigated in the context of psychological disorders, especially anxiety^28–31^. In mice, a knockout of *HTR1A* led to an increase in anxiety-related behavior^32^, while in transgenic mice the upregulation of the *HTR1A* gene induced less anxiety-like behavior^33^. 5-HT_1A_ receptors are either pre-synaptic somatodendritic autoreceptors or post-synaptic heteroreceptors. While alterations of both are linked to anxiety disorders and depression, especially heteroreceptors are downregulated in individuals with mental disorders, implying anxiety disorders, as reviewed in Albert^30^. In the context of the amygdala transcriptome, *HTR1A* and *HTR2A* have been described to be involved in fear memory^34^, where *HTR1A* was downregulated as well when animals were exposed to physical stressors^35^. In contrast to our results, where *HTR2A* was downregulated in the amygdala of the animals that were stunned with CO_2_, *HTR2A* was upregulated in the amygdalae of mice that had experienced physical stressors^35^. One explanation for these different expression profiles could be a different reaction to innate and learned fear. Isosaka et al*.*^36^ showed that *HTR2A*-expressing cells are differently involved in the reaction to these different types of fear. Whether this relationship applies to the up- and down-regulation of the gene itself cannot be proven with the data presented here. Besides the described genes of the serotonin pathway, the *WNT2* gene expression was also downregulated in animals stunned with CO_2_ (Fig. 2c, Supplementary Information S1, S2). Zhou et al*.*^37^ showed that the expression of *WNT2* in the hippocampus of mice was downregulated when they were forced to undergo chronic restrain stress. An increase in *WNT2* expression resulted in less depression-like behavior but had no effect on anxiety-like behavior. It is possible that different inert gases that induce unconsciousness via hypoxia have different effects on processes in the amygdala. In our data set, 111 or 30 genes were found to be significantly differentially expressed with respect to the applied p-value threshold. One gene that was differentially upregulated in Ar but not in N_2_ was *NPS*. *NPS* encodes Neuropeptide S, which is known to be a regulator of fear and anxiety ^38^, as it is an anxiolytic compound in mice. Intracerebroventricularly administration of Neuropeptide S in mice increased the time in open arms in an elevated plus maze test and decreased marble burying^39^. Therefore, it is possible that pigs stunned with Ar had less aversive experiences than those stunned with CO_2_ and also less than those stunned with N_2_. Nevertheless, it has to be taken into account that gene expression data provides an initial insight into affected processes. However, the extent to which information is lost during the translation process ^40^ cannot be estimated in the current study. Further investigations on a histological basis may assist to provide deeper insights.^32,34^ Additionally, with the presented data, it remains unknown which processes are actively perceived by the pig during stunning reaching unconsciousness. While our findings provide initial insights, future studies in other brain regions, like the prefrontal cortex^41^ as well as investigations of additional stress-related pathways^42^, may provide a more comprehensive understanding of the processes in pigs.

Our findings underline previous reports of aversive behavior of pigs stunned with CO_2_ by providing the first whole transcriptome study of the amygdalae from pigs stunned with different gas mixtures. We showed that in the case of inert gas stunning less anxiety-related genes were expressed in the amygdala of the pigs.

## Material and methods

### Animals and stunning

A total of 27 female and castrated male crossbreed pigs ([Danish Landrace x Danish Yorkshire]x Pietrain) were examined in this study. These 27 pigs were a subset of 1300 pigs, slaughtered on 18 experimental days, which were part of the project for “Testing inert gases in order to establish replacements for high concentration CO_2_ stunning for pigs at the time of slaughter (TIGER)”^43^. Out of 54 pigs slaughtered on a single experimental day, 27 animals were selected for amygdala transcriptome analysis. For each gas mixture treatment, the first nine animals at the slaughter line were sampled. All pigs originated from the same farm. The average slaughter weight was 96.7 ± 7.2 kg. The pigs arrived at the slaughterhouse in the morning and were stunned and slaughtered between 18:30 and 21:45. All pigs were handled according to Council Regulation (EC) No 1099/2009^4^, with specifications according to the German legislation^44^. All animal handling was carried out by slaughterhouse personnel, who were instructed to handle the animals as gently as possible. The animals were equally distributed to one of the used gas mixtures, which was either a) 95% Argon (Ar), b) a nitrogen – Ar mixture (N_2_) with 51% N_2_ and 48% Ar in the mixture or c) more than 90% CO_2_. Stunning was carried out in a Dip-Lift-System in a commercial slaughterhouse, which was equipped with a new and patented gassing technology by Air Liquide Deutschland GmbH (Krefeld, Germany) to achieve the required inert gas concentrations. With this technology a residual oxygen concentration  ≤ 1.03% was achieved for all gas mixtures used for stunning. Each gondola was loaded with two pigs. Dwell time was 180 s for CO_2_, 240 s for Ar and 250 s for N_2_. After stunning and exsanguination the carcasses entered the normal slaughter routine of the abattoir.

### Sample collection

After evisceration and splitting, the brains of the animals were removed and collected. A tissue block of approximately 25 mm^3^ was excised from the amygdala 30 min *post mortem* and stored directly in RNA*later* ™ (Thermo Fischer Scientific, Waltham, Massachusetts, USA) solution. The locations of amygdalae were identified with the “Stereotaxic Atlas of the Pig Brain”^45^.

### RNA isolation

Total RNA isolation was performed using the RNeasy Plus Universal Mini Kit (Qiagen N.V., Hilden, Germany). Approximately 20 mg of frozen brain tissue was transferred into a 2 ml tube and mixed with 900 µl QIAzol Lysis Reagent (Qiagen N.V., Hilden Germany) and 5 µl Reagent DX (Qiagen N.V., Hilden, Germany). Additionally, 1.4 mm Ceramic Beads (Biolabproducts GmbH, Bebensee, Deutschland) were added to the mixture, and tubes were loaded into a Bead Ruptor Elite (Omni International, Kennesaw GA, USA) and processed in 3 cycles with 4.5 m/sec for 15 s and a dwell time for 10s. Afterwards, the samples were processed according to the manufacturer´s specifications and eluted in RNAse free water.

### RNA sequencing

RNA sequencing was performed as a service by BGI genomics. Library type was DNBSEQ Eukaryotic Strand-specific mRNA library and sequencing was performed with a DNBseq platform. Paired-end reads with a read length of 100 base pairs were produced. Raw sequencing reads were filtered and trimmed with SOAPnuke with the following settings: *-n 0.001 -l 20 -q 0.4 –adaMR 0.25 –ada_trim –minReadLen 100*.

### Transcriptome analysis

Pig sequencing reads were aligned to the *Sus scrofa* 11.1 reference genome version GCF_000003025.6 and mice sequencing reads were aligned to the GRCm39 reference genome version GCF_000001635.27 using HiSat2 version 2.1.0 with default settings^46^. Splice sites were derived from the Gene transfer format (GTF) file. FeatureCounts from the Subread package (Version 2.0.0) was used to count exon spanning reads^47^. Differential expression (DE) analyses were conducted with DESeq2 (Version 1.40.1)^48^. The R package Enhanced Volcano (Version 1.18.0) was used to generate volcano plots of DE results.

### Functional annotation

Gene cluster comparison and visualization were achieved with the R package clusterProfiler (Version 4.8.1)^49^. Gene symbols were converted to ensemble IDs with the clusterProfiler Biological Id Translator (bitr). Gene ontology (GO) term analyses were performed with enrichGO (settings: pAdjustMethod = “fdr”, pvalueCutoff = 1, qvalueCutoff = 0.25, readable = TRUE, minGSSize = 10). KEGG pathway analysis was done with enrichKEGG (settings: pvalueCutoff = 1, pAdjustMethod = "BH “, minGSSize = 10, maxGSSize = 500, qvalueCutoff = 0.25, use_internal_data = FALSE). Plots were created with the dotplot function. The heatmap was created with the R package heatmaply (Version 1.4.2). Protein–protein interaction maps were constructed with StringDB (Version 11.5)^50^.

## Supplementary Information

Supplementary Information 1.

Supplementary Information 2.

Supplementary Information 3.

Supplementary Information 4.

Supplementary Information 5.

Supplementary Information 6.

## Data Availability

Raw sequencing data will be released upon acceptance of the manuscript. The data is accessible via this link https://www.ncbi.nlm.nih.gov/bioproject/PRJNA1177806
