# Supplementary material for: Carbon dioxide stunning of pigs induces the expression of fear-associated genes in the amygdala
Source: Sci Rep. 2026 May 6;16:14416. doi: 10.1038/s41598-026-51710-9 (PMC13149842; doi:10.1038/s41598-026-51710-9)

Additional File S5: Protein protein interaction (PPI) analysis results. (A) DEGs between Ar vs CO_2, (B) Ar vs N_2, and (C) CO_2 vs N_2.

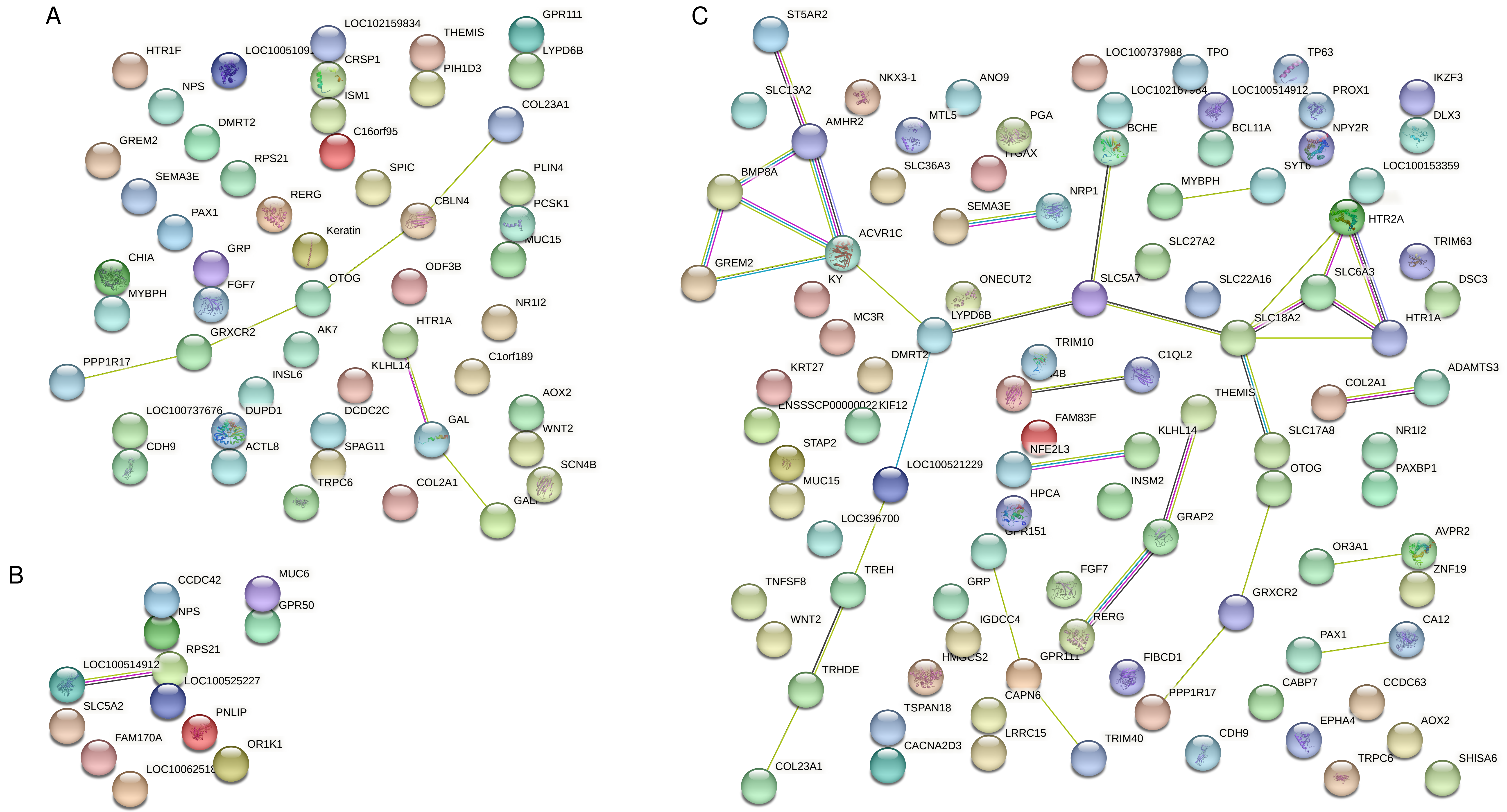

Supplement: Supplementary file 5 — Supplementary Information 5. [file 41598_2026_51710_MOESM5_ESM.pdf]
